# Progressive Versus Conservative Chest Tube Management Following Surgery for Primary Spontaneous Pneumothorax

**DOI:** 10.1093/ejcts/ezag136

**Published:** 2026-03-26

**Authors:** Quirine C A van Steenwijk, Marcel G W Dijkgraaf, Wolter Oosterhuis, Denis Susa, Matthijs D M Bolmers, Robert T J Kortekaas, Chris Dickhoff, Jerry Braun, Frank J C van den Broek

**Affiliations:** Department of Surgery, Maxima Medical Center, 5504 DB Veldhoven, Netherlands; Department of Epidemiology and Data Science, Amsterdam UMC, 1105 AZ Amsterdam, The Netherlands; Methodology, Amsterdam Public Health, University of Amsterdam, 1105 AZ Amsterdam, The Netherlands; Department of Surgery, Haaglanden Medical Center, 2262 BA Leidschendam, The Netherlands; Department of Surgery, Bravis Hospital, 4624 VT Bergen op Zoom, The Netherlands; Department of Surgery, Dijklander Hospital, 1624 NP Hoorn, The Netherlands; Department of Surgery, Franciscus Gasthuis, 3045 PM Rotterdam, The Netherlands; Department of Cardiothoracic surgery, Amsterdam UMC, Vrije Universiteit Amsterdam, 1081 HV Amsterdam, The Netherlands; Department of Cardiothoracic Surgery, Leiden University Medical Center, 2333 ZA Leiden, The Netherlands; Department of Surgery, Maxima Medical Center, 5504 DB Veldhoven, Netherlands

**Keywords:** primary spontaneous pneumothorax, chest tube management, VATS

## Abstract

**Objectives:**

“Enhanced recovery after thoracic surgery” is increasingly implemented following surgery for primary spontaneous pneumothorax (PSP). Historically, postoperative chest tubes are left in place for several days to ensure adequate pleurodesis and prevent recurrence. Prompt chest tube removal upon cessation of air leakage may enhance recovery but potentially increases risk of recurrence. We evaluated progressive chest tube management following surgical pleurodesis for PSP.

**Methods:**

Retrospective multicentre study of patients undergoing surgical pleurodesis for PSP between 2020 and 2023. Hospitals with implemented progressive chest tube management (removal upon cessation of air leakage <8 hours) were compared against hospitals with conservative management (removal after 1-2 days). Outcome measures, corrected for bias using directed acyclic graphs, were postoperative length of stay (LOS), chest tube duration, complications, and ipsilateral recurrences.

**Results:**

A total of 183 patients from 5 hospitals were included: 99 patients in the progressive and 84 in the conservative group. Follow-up and baseline characteristics were comparable, except for surgical indication, pleurodesis technique, and use of postoperative chest X-ray. Progressive management significantly reduced LOS (Δ1 day), chest tube duration (Δ2 days), and complications (9.1 vs 21.4%). After correction for pleurodesis technique, persistent air leakage, and chest X-ray, LOS was reduced by 1.03 day (*P* < .001). Recurrences (6.6%) were similar across groups (*P* = .388).

**Conclusions:**

Progressive chest tube management reduces LOS, chest tube duration, and complications without increasing the risk of recurrence in this retrospective comparative study. A prospective study is necessary to validate these findings before considering widespread implementation.

## Graphical abstract

**Figure:**
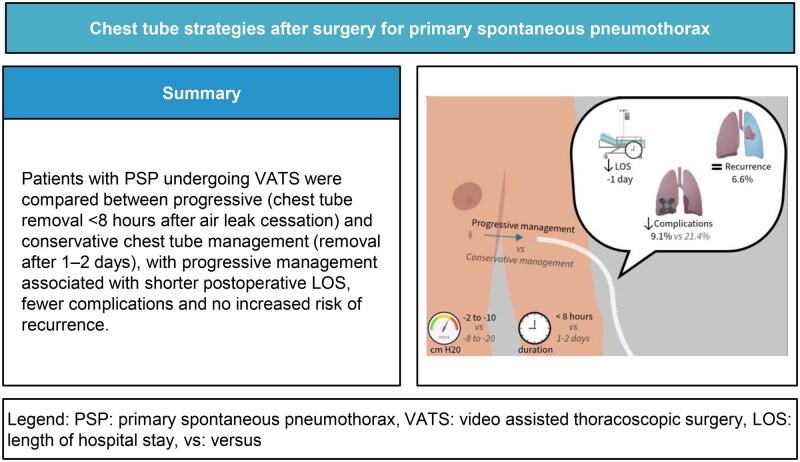

## Introduction

Surgical pleurodesis is the preferred treatment in case of recurrent or persistent primary spontaneous pneumothorax (PSP), also to reduce the risk of further recurrence.^1^ Pleurodesis may be performed by either mechanical or chemical pleurodesis, followed by chest tube drainage, to facilitate re-expansion and coalescence of the pleura. Historically, chest tubes were inserted for at least 3-5 days, regardless of air leakage, to assure adequate pleurodesis and to prevent further recurrence. Enhanced recovery after thoracic surgery (ERATS) principles have encouraged the employment of early chest tube removal in case of absence of air leakage for 6-12 hours and fluid production <450-500 cc/24 hours.^2^^,^^3^ Early chest tube removal may reduce pain and enhance recovery with shortened length of stay (LOS).^4^^,^^5^

Besides chest tube duration, the ERATS guideline also reports that type of analgesic technique may substantially influence LOS.^2^ Whereas thoracic epidural analgesia (TEA) is associated with immobilization, urinary retention, and hypotension, loco-regional analgesic techniques such as single-shot intercostal or paravertebral block may still provide sufficient pain control but accelerate recovery.^6^^,^^7^

So far, only 3 single-arm single-centre studies reported on early chest tube removal combined with loco-regional analgesia after surgery for PSP.^8–10^ The results of these studies suggest the safety of progressive chest tube management with a LOS of only 1 day. However, direct comparative studies against a more conservative approach are lacking, which hinders widespread implementation of progressive chest tube management.

We previously reported on the large variability in perioperative care concerning chest tube removal and analgesic techniques among Dutch thoracic surgeons.^11^ Dutch hospitals claiming progressive chest tube and pain protocols after video-assisted thoracic surgery (VATS) pleurodesis still harboured differences in their chest tube removal criteria and were invited to participate in this retrospective comparative study. The aim of this study was to compare outcomes of hospitals with progressive chest tube management against hospitals with a more conservative approach after VATS pleurodesis for PSP.

## Patients and methods

### Ethics statement, hospital selection, and study period

After approval from the institutional review board, retrospective data were collected from 5 hospitals in the Netherlands. Explicit informed consent was waived; however, medical records were only reviewed in case patients did not refuse to participate in medical research in advance. Hospitals were selected based on their claimed progressive postoperative chest tube and pain management, resulting from a previous national survey.^11^ Progressive management was defined as both early postoperative chest tube removal and the use of intraoperative multilevel single-shot intercostal analgesia, with anaesthetic injection adjacent to the sympathetic chain ensuring both paravertebral and intercostal visual spread. Next to intercostal analgesia, multimodal analgesic regimen was maintained consisting of paracetamol, non-steroidal anti-inflammatory drugs and short-acting morphine. In cases of insufficient pain relief, additional long-acting morphine was administered orally or intravenously.

Once progressive management was fully implemented at each site (ranging from 2020 to 2022; **Table 1**), all subsequent eligible patients were included. Hospital-specific protocols mandate surgical pleurodesis, with each hospital employing either near-total pleurectomy or talcage of the entire thoracic cavity (dispersion of one vial Steritalc of 3 g) exclusively.

**Table 1.** (Peri)operative In-house Protocol Per Hospital, Differentiating Progressive (Removal at 4-8 Hours) and Conservative Approach (Removal at 1-2 Days)

Hospital	Date of start inclusion^a^	*N*	Pleurodesis technique	Routine postoperative chest X-ray	Thopaz setting	Postoperative chest tube removal criteria
Progressive approach						
#1	June 2022	6	Pleurectomy	Yes	−2 cmH_2_O	0 mL/min air leakage or bloody drainage for 4 hours
#2	June 2020	57	Talcage	No	−2 or −4 cmH_2_O	<20 mL/min air leakage and <100 mL fluid drainage for 6 hours
#3	January 2020	36	Talcage	Yes	−10 cmH_2_O	<30 mL/min air leakage and <100 mL fluid drainage for 8 hours
Conservative approach						
#4	January 2020	34	Pleurectomy (*n* = 21) or Talcage (*n* = 13)	Yes	−8 to −10 cmH_2_O	0 mL/min air leakage at 2 days
#5	February 2020	50	Talcage	Yes	−10 to −20 cmH_2_O	0 mL/min air leakage and fluid drainage <400 mL/d at 1 or 2 days depending on perioperative findings

a The inclusion period started once single-shot intercostal nerve blockade was implemented as routine analgesic technique in each study centre.

Abbreviation: N: number of patients.

### Patient selection criteria

From the start of inclusion until December 2023, all consecutive patients who underwent VATS pleurodesis for PSP were eligible. Exclusion criteria were secondary spontaneous pneumothorax, pleurodesis for other indications, previous ipsilateral thoracic surgery, pleurodesis by both pleurectomy and talcage, and insufficient data (eg, no surgical report). For patients with PSP, the surgical indications were recurrent PSP, a first episode of PSP with persistent air leakage (PAL) lasting more than 5 days, or patient preference. Both electively planned and emergent operations were included.

### Postoperative chest tube management

It was standard practice to place one postoperative chest tube (size 20-28 Ch). All hospitals used the same digital chest drainage system (Thopaz+, Medela, Switzerland) with standardized in-house protocols throughout the entire study period. However, different settings and chest tube removal criteria were applied in each hospital by all surgeons, categorizing hospitals into *progressive* or *conservative* regarding their chest tube management (**Table 1**). Progressive management was defined as a Thopaz setting between −2 to −10 cmH_2_O and chest tube removal within 4-8 hours postoperatively, provided that air leakage had ceased. Conservative management was defined as a Thopaz setting between −8 to −20 cmH_2_O and chest tube removal no earlier than 24-48 hours after surgery, once air leakage had stopped.

### Outcome measures

The primary outcome measure was postoperative LOS in days. All hospitals applied the following general discharge criteria: absence of chest tube, sufficient pain control with oral analgesics, and being able of self-care. Secondary outcomes were postoperative chest tube duration (days), complication rates within 30 days after surgery, ipsilateral recurrences, and time-to-recurrence during follow-up. Complications were categorized as either minor (Clavien-Dindo 1-2) or major (Clavien-Dindo ≥3 and/or PAL >5 days).^12^ If patients experienced more than one complication, the highest Clavien-Dindo classification was scored. Patients had a postoperative appointment at the outpatient clinic within 1 month. Hereafter, patients were advised to contact the hospital in case of symptoms suspicious for recurrence. Follow-up was defined as time from the date of surgery until the date of final check of the medical record. All ipsilateral recurrences were radiographically confirmed and was defined as either a new or progressive pneumothorax on chest X-ray after chest tube removal, compared with a prior chest X-ray, or, when routine postoperative chest X-ray was not performed, on chest X-ray obtained in response to patient-reported symptoms demonstrating recurrence.

### Statistical analysis

Outcomes were compared between patients from hospitals with progressive management and those from hospitals with conservative management. Results were analysed using Statistical Package for Social Sciences (SPSS, version 22.0, IBM, Armonk, NY). Categorical baseline characteristics were compared using the chi-square test, and continuous variables were analysed using the independent t-test, 1-way ANOVA, Mann-Whitney U, or Kruskal-Wallis test when appropriate. The Kolmogorov-Smirnov test was used to assess the similarity of distributions. Continuous variables were expressed as mean with standard deviation (normal distribution) or median with interquartile range (IQR) (non-normal). Categorical variables were expressed as number with percentages (%). Mean time-to-recurrence with 95% confidence interval (95% CI) was analysed using the Kaplan-Meier method, and the log-rank test was used to assess differences between groups. Censoring occurred at the date of final check of the medical record. All variables were assessed for missing values, which are presented in **Tables 2** and **3**. In case of less than 5% missing values, no imputation was applied, as the potential impact was expected to be minimal. If any variable had more than 5% missing data, multiple imputation will be performed under the assumption of missing at random.

**Table 2.** Patient and Surgical Characteristics for the Conservative and Progressive Chest Tube Groups

		Total *n* (%)	Conservative *n* (%)	Progressive *n* (%)	*P-*value
Patient characteristics					
Patients	Total	183	84	99	
Per hospital	#1	6 (3.3)	0	6 (6.1)	
	#2	57 (31.1)	0	57 (57.6)	
	#3	36 (19.7)	0	36 (36.4)	
	#4	34 (18.6)	34 (40.5)	0	
	#5	50 (27.3)	50 (59.5)	0	
Gender	Male	161 (88.0)	75 (89.3)	86 (86.9)	.616
ASA	1	53 (29.0)	24 (28.6)	29 (29.3)	.754
	2	114 (62.3)	52 (61.9)	62 (62.6)	
	3	15 (8.2)	7 (8.3)	8 (8.1)	
	4	1 (0.5)	1 (1.2)	0	
Smoking history^a^	Never	55 (30.1)	25 (29.8)	30 (30.3)	.258
	Current	75 (41.0)	29 (34.5)	46 (46.5)	
	Former	46 (25.1)	26 (31.0)	20 (20.2)	
	Missing	7 (3.8)	4 (4.8)	3 (3.0)	
Age, years	Median (IQR)	26.0 (20.0-42.0)	29.0 (21.3-43.0)	25.0 (20.0-42.0)	.563
Surgical indication	First episode PSP^b^	60 (32.8)	36 (42.9)	24 (24.2)	.008
	Recurrent PSP	123 (67.2)	48 (57.1)	75 (75.8)	
Preoperative chest tube present	Yes	91 (49.7)	46 (54.8)	45 (45.5)	.226
	Missing	1 (0.5)	1 (1.2)	0	
Surgical characteristics					
VATS	Single port	31 (16.9)	11 (13.1)	20 (20.2)	.442
	Multiportal	150 (82.9)	72 (85.7)	78 (78.8)	
	Missing	2 (1.1)	1 (1.2)	1 (1.0)	
Bullectomy	Bullectomy	154 (84.2)	75 (89.3)	79 (79.8)	.148
	Apical wedge resection	19 (10.4)	7 (8.3)	12 (12.1)	
	No bullectomy	10 (5.5)	2 (2.4)	8 (8.1)	
Type of pleurodesis	Pleurectomy	27 (14.8)	21 (25.0)	6 (6.1)	<.001
	Talcage	156 (85.2)	63 (75.0)	93 (93.9)	
Duration of surgery, min	Median (IQR)	39.0 (31.5-49.5)	38.0 (30.0-48.3)	42.0 (32.0-50.0)	.422
	Missing	2 (1.1)	2 (2.4)	0	
Thopaz setting, cmH_2_O	Median (IQR)	−10 (−10 to −4)	−10 (−15 to −10)	−4 (−10 to −2)	<.001
Routine chest X-ray	No	54 (29.5)	6 (7.1)	48 (48.5)	<.001
	Yes	128 (70.5)	78 (92.6)	51 (51.6)	
	fully expanded lung	50 (39.1)	34 (43.6)	16 (31.4)	
	not fully expanded lung	78 (60.9)	44 (56.4)	35 (68.6)	

For all variables without displayed missing values, the dataset is complete.

a Smoking history consisted of smoking cigarettes and/or cannabis.

b First episode PSP includes also patients >5 days of air leak.

Abbreviations: ASA, American Association of Anesthesiologists; IQR, interquartile range; n, number of patients; PSP, primary spontaneous pneumothorax; VATS, video-assisted thoracoscopic surgery.

**Table 3.** Primary and Secondary Outcomes for the Conservative and Progressive Chest Tube Groups

		Total *n* (%)	Conservative *n* (%)	Progressive *n* (%)	*P*-value
Primary outcome					
Length of stay, days	Median (IQR)	3.0 (2.0-4.0)	3.0 (3.0-4.0)	2.0 (1.0-3.0)	<.001
Secondary outcomes					
Chest tube duration, days	Median (IQR)	2.0 (1.0-3.0)	3.0 (2.0-3.0)	1.0 (1.0-2.0)	<.001
Complications	Yes	27 (14.8)	18 (21.4)	9 (9.1)	.019
	Minor^a^	13 (7.1)	9 (10.7)	4 (4.0)	
	Major^b^	14 (7.7)	9 (10.7)	5 (5.1)	
Type of complications^c^	PAL > 5 days	13 (7.1)	8 (9.5)	5 (5.1)	
	Bleeding	3 (1.6)	3 (3.6)	0	
	Infection^d^	5 (2.7)	5 (6.0)	0	
	Urinary retention	5 (2.7)	5 (6.0)	0	
	Other^e^	7 (3.8)	3 (3.6)	4 (4.0)	
Recurrences	Ipsilateral	12 (6.6)	7 (8.3)	5 (5.1)	–
Time to recurrence, days	Mean (95% CI)	51.5 (49.6-51.8)	48.8 (45.9-51.8)	52.2 (49.9-54.6)	.388^f^
Treatment recurrence	Wait-and-see	1 (8.3)	1 (14.3)	0	–
	Admission for observation	1 (8.3)	0	1 (20.0)	–
	Chest tube	6 (50.0)	2 (28.6)	4 (80.0)	–
	Surgery	4 (33.4)	4 (57.1)	0	–
Follow-up, months	Median (IQR)	30.0 (19.0-44.0)	33.0 (22.0-43.0)	29.0 (19.0-45.0)	.778

a Clavien-Dindo score 1 or 2.

b Clavien-Dindo score of ≥3 and/or persistent air leakage (PAL) >5 days.

c Some patients suffered more than 1 complication.

d Infection included postoperative pneumonia (*n* = 2) and wound infection (*n* = 3).

e Other complications included cardiac arrhythmia (*n* = 1), anaphylactic reaction on medication (*n* = 2), delirium (*n* = 1), neuropraxia (*n* = 3).

f Log rank test. Abbreviations: IQR, interquartile range; N, number of patients. For all variables without displayed missing values, the dataset is complete.

To determine the true effect of progressive versus conservative chest tube management on the outcome measures of interest, directed acyclic graphs (DAGs) were employed to identify variables that potentially introduce bias. Using DAGs, both causal and non-causal pathways between chest tube management and the outcome measures were mapped, enabling the identification of variables that might bias the association through confounding or effect modification.^13^ The reported graphs are based on current clinical knowledge and may be refined in future studies as new evidence emerges. The identified potential confounders or effect modifiers were subsequently tested by multivariable linear or logistic regression analysis. Variables were regarded as confounder if the regression coefficient (β) of chest tube management as independent variable changed ≥|10%| and as effect modifier if the interaction term had a *P*-value <.05.^14^ Subsequently, confounders and effect modifiers were incorporated into a multivariable regression model. The model’s validity was assessed by evaluating the presence of outliers (standardized residuals outside ±3 SDs), the independence of errors (Durbin-Watson test [±2]), normality assumptions, and the correlation coefficient between the observed and predicted values of the dependent variable (*R*^2^).^15^ Although DAGs prevent correcting for variables in the same pathway, the final model was additionally tested for collinearity (variance inflation factor >5) which was accepted only in case it existed between the variable and its interaction term. In the presence of multiple outliers, the natural logarithm of the dependent variable was applied to normalize the distribution. The results were converted back with Euler’s number (e^*x*^). The tested outcome measures were LOS, complications and recurrence (**Figure S1A**-**S1C**). Statistically significance was considered at *P* < .05.

## Results

**Figure 1.**
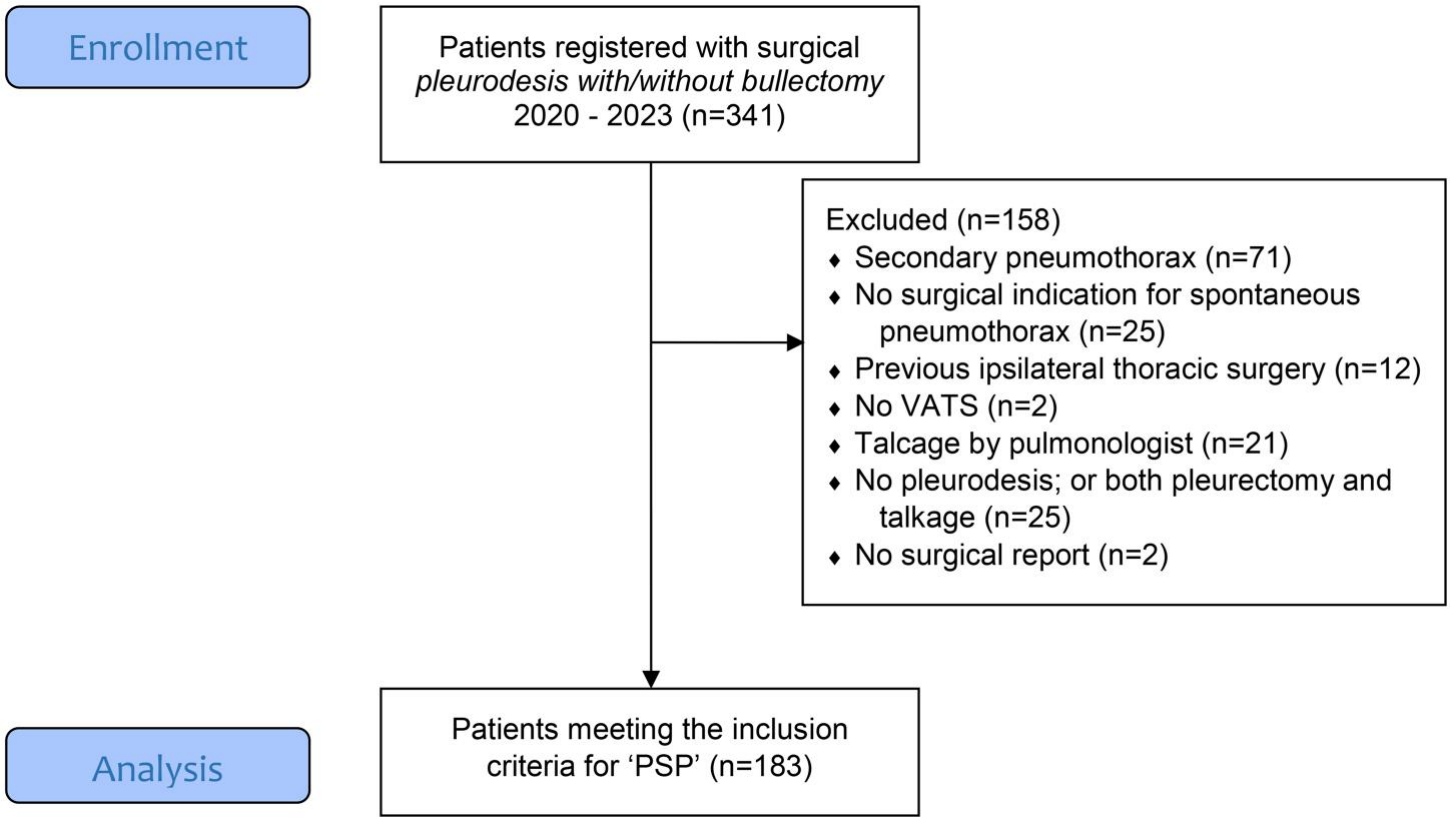
CONSORT 2010 Flow Diagram of Patient Selection. Abbreviations: N, number; PSP, primary spontaneous pneumothorax; VATS, video-assisted thoracic surgery.

In the study period, 359 patients underwent VATS pleurodesis across the 5 included hospitals. After excluding ineligible patients, 183 PSP patients remained for our analysis (**Figure 1**). Median age was 26 (IQR 20-42) years and 88% was male. Based on the existing in-house chest tube protocols (**Table 1**), 99 patients were identified in the progressive and 84 patients in the conservative group. Baseline characteristics are presented in **Table 2**. One of the conservative hospitals (#5) also performed surgery following a first episode of PSP, irrespective of preoperative PAL, accounting for an overrepresentation of this surgical indication in the conservative group, *P* = .008. Likewise, due to the late de-implementation of TEA (until June 2022) at one progressive hospital (#1, utilizing pleurectomy), 12 patients from this hospital were not eligible for inclusion, resulting in an overrepresentation of “pleurectomy” as pleurodesis technique in the conservative group. Finally, one conservative hospital did not conduct postoperative chest X-rays routinely.

### Primary outcome

The median LOS was significantly shorter in the progressive group compared to the conservative group, with 2.0 days (IQR 1.0-3.0) versus 3.0 days (IQR 3.0-4.0) (*P* < .001), respectively (**Table 3**). With respect to the validity assumptions of linear regression, the natural logarithm of LOS was used in the multivariable linear regression analysis to normalize the distribution. The routine use of postoperative chest X-ray acted as confounder (Δβ = 42%; *P* < .001; **Table S1**) prolonging LOS, whereas pleurodesis technique (*P* = .001) and PAL (*P* = .014) were identified as effect modifier. The final model including chest X-ray (yes or no), pleurodesis technique (pleurectomy or talcage) and PAL (yes or no) is presented in **Table 4**. The model resulted in standardized residuals of 3.4, a Durbin Watson of 1.99 with an *R*^2^ of 64.4% and VIF <5. After correction of confounding and effect modification, the LOS in the progressive group was 1.03 days shorter compared to the conservative group (*P* < .001). In **Table S2**, the logarithmic LOS is transformed to the original scale to illustrate the potential impact on LOS for different scenarios comparing progressive versus conservative chest tube management.

**Table 4.** Relation Between Progressive Chest Tube Management and Natural Logarithm of Length of Stay After Correction for Effect Modification and Confounding

LOS	β	95% CI	*P*-value	Difference in days^a^
*Univariable*				
*Constant*	1.931	1.654 to 2.209	<.001	
Chest tube management	−0.634	−0.805 to −0.462	<.001	
Progressive management				−1.72
*Multivariable*				
*Constant*	2.085	1.319 to 2.850	<.001	
Chest tube management	−1.087	−1.587 to −0.588	<.001	
Progressive management				−1.03
Chest X-ray	0.429	0.275 to 0.583	<.001	
With postoperative X-ray				+1.06
PAL	0.523	−0.185 to 1.231	.147	
Interaction management*PAL	0.548	0.068 to 1.028	.025	
PAL present				+7.14
Pleurodesis technique	−0.745	−1.294 to −0.196	.008	
Interaction management*pleurodesis	0.585	0.176 to 0.995	.005	
Pleurectomy				+0.45

Chest tube management: conservative = 1 and progressive = 2. Postoperative chest X-ray: no = 0 and yes = 1.

Air leakage > 5 days: no = 0 and yes = 1. Pleurodesis technique: talcage = 1 and pleurectomy = 2.

a The difference in days per variable being present or not is calculated on the normal scale.

Abbreviations: β, regression coefficient; CI, confidence interval; LOS, length of stay; PAL, persistent air leakage.

### Secondary outcomes

**Figure 2.**
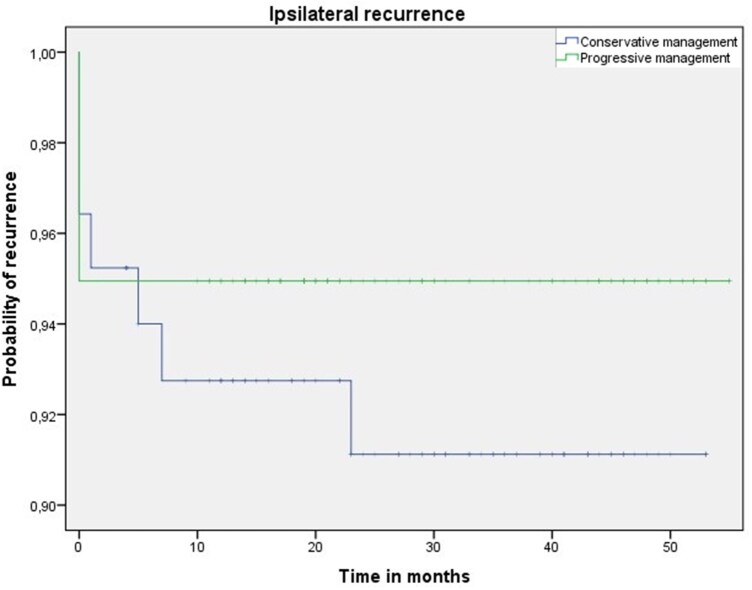
Ipsilateral Recurrences Over Time After Progressive and Conservative Chest Tube Management. Kaplan-Meier curve displaying the estimated recurrence probability for the conservative (blue) and the progressive (green) groups. Each vertical step in the curve indicates one or more ipsilateral recurrences and censored patients are indicated by a vertical mark in the curve at the censoring time. The log-rank test has a *P*-value of .402.

The median chest tube duration was 1.0 day (IQR 1.0-2.0) in the progressive versus 3.0 days (IQR 2.0-3.0) in the conservative group (*P* < .001; **Table 3**). The overall complication rate was 9.1% in the progressive versus 21.4% in the conservative group (*P* = .019). Only minor complications were present in 4.0% versus 10.7%, respectively, whereas 5.1% vs 10.7% harboured also major complications. The most reported complications yielded PAL, urinary retention, postoperative bleeding, and infection. No factors identified by the DAG were causing bias between progressive chest tube management and complications (**Figure S1B** and **Table S1**). Ipsilateral recurrences occurred in 7 patients (8.3%) in the conservative versus 5 (5.1%) in the progressive group during the median follow-up of 33 months (IQR 22-43) versus 29 months (IQR 19-45) respectively (*P* = .778). Routine postoperative chest X-ray was performed in 11 of the 12 patients with recurrence, allowing confirmation that the observed recurrences represented true events rather than residual apical spaces or incomplete postoperative lung expansion. In one patient, an ipsilateral recurrence was diagnosed on a postoperative chest X-ray, which was obtained in response to symptoms. The mean time to recurrence was 48.8 months (95% CI, 45.9-51.8) in the conservative group and 52.2 months (95% CI, 49.9-54.6) in the progressive group (log-rank *P* = .388) (**Table 3** and **Figure 2**). Treatment of recurrences did not differ between the groups and varied between a wait-and-see approach (with or without hospital admission), chest tube insertion and redo-surgery. No factors identified by DAG were causing bias between progressive chest tube management and recurrence (**Figure S1C** and **Table S1**).

## Discussion

This multicentre study revealed that hospitals implementing progressive chest tube management, comprising early removal and low-vacuum Thopaz setting, achieved significantly shorter LOS and chest tube duration, fewer complications, and comparable recurrences rates following VATS pleurodesis for PSP.

Due to the retrospective setting of our study, several other perioperative factors may have played a crucial role next to the settings of the digital drainage system and the timing of chest tube removal. Type of pleurodesis technique and PAL were effect modifiers, and routine postoperative conduction of chest X-ray was a confounder for LOS. Routine conduction of chest X-rays was associated with prolonged LOS, probably due to the additional time required to obtain and interpret X-rays, or due to increased hesitation among clinicians to remove chest tubes in case of a non-fully expanded lung. Although most Dutch surgeons routinely use postoperative radiographs, others recommend against it since they provide no added value compared to radiographs based on clinical indication alone.^16^ Conducting a postoperative chest X-ray serves to confirm adequate lung expansion following surgery, although this may alternatively be assessed intraoperatively prior to closure. One of our included progressive hospitals conducted postoperative chest X-rays only in response to clinical complaints and not routinely, explaining the slightly higher observed proportion of not fully expanded lungs in the progressive group (68.6% versus 56.4%). Whereas guidelines lack recommendations on postoperative radiographs, our results suggest that its routine use may prolong LOS.^1^^,^^2^

In line with our expectations, the median chest tube duration was significantly shorter by 2 days in the progressive group. Although median chest tube duration in the progressive group was 1 day, the median LOS remained 2 days, indicating that factors beyond the presence of a chest tube also influence hospital discharge. Three retrospective studies focusing on chest tube removal on the same day of surgery showed similar findings regarding LOS.^8–10^ The ERATS guideline outlines 44 additional items that enhance recovery and impact LOS.^2^ One of the key factors is pain management. We previously demonstrated that single-shot intercostal nerve block for postoperative pain management was associated with higher pain scores compared to TEA until the second postoperative day.^7^ This likely contributed to the observed discrepancy between chest tube duration and LOS in our study, which included only patients receiving single-shot analgesia. Consistent with this reasoning, chest tube duration and LOS in the conservative group were comparable at 3 days, coinciding with the time when pain scores between TEA and intercostal nerve block have equalized.^5^

We also found the progressive approach to be accompanied by a 12% reduction in complications when compared with the conservative approach, predominantly due to lower rates of infection, urinary retention and PAL. Early chest tube removal facilitates early mobilization and limits the duration of foreign body presence, potentially reducing urinary retention and the risk of infection. Also, the lower vacuum setting of the digital drainage system in the progressive group may have contributed to the reduction in PAL. A randomized trial demonstrated that low suction levels were associated with decreased air leakage and fluid drainage in lobectomy cases.^17^ A retrospective study among pleurodesis cases showed the same findings.^18^

Whereas the observed complication rate in the conservative group aligns with data from the Dutch national registry,^19^ complications were notably lower in the progressive group. Critics may suggest that the more frequent use of talcage in the progressive group contributed to the reduced complication rate (eg, reduced risk of bleeding). As we also hypothesized talcage to be associated with a lower risk of postoperative bleeding, and consequently a reduced LOS, type of pleurodesis was incorporated as potential effect modifier in our DAG. Interestingly, all cases of postoperative bleeding, infection, and urinary retention occurred in patients who underwent talcage, resulting in an unexpected but overall increasing effect on LOS. However, the small number of events and limited number of patients who underwent pleurectomy prevented us from an in-depth analysis on complications.

Finally, prolonged chest tube insertion (eg, 3-5 days) was historically considered essential to achieve adequate pleurodesis and thereby prevent recurrence. As progressive chest tube management might therefore theoretically result in higher recurrence rates, our findings indicate its safety, with observed recurrence rates of 5.1% in the progressive versus 8.3% in the conservative group. Also time-to-recurrence was comparable between the 2 groups. Contrary to our findings, Furuya et al^9^ reported a higher recurrence rate of 11.4% following early chest tube removal but did not provide details on the onset of recurrences. Possible explanations include variations in surgical pleurodesis technique and inclusion criteria (eg, previous ipsilateral surgery). Regarding pleurodesis technique, 2 meta-analyses favoured chemical pleurodesis in terms of recurrence, although recommendations were flawed due to lack of high-quality studies and inconclusive results.^20^^,^^21^ The recurrence rates observed in both the progressive and conservative groups fell within the range reported in the literature and no association between pleurectomy or talcage and recurrence was identified.

Although this is the first study directly comparing progressive against conservative chest tube management in PSP, our study has some limitations. In addition to Thopaz setting and timing of chest tube removal, we observed other differences between hospitals, such as the use of chest X-ray and type of surgical pleurodesis. Although we adjusted for these variables, they may still be considered integral components of a progressive chest tube strategy and represent a part of a broader institutional care culture, indicating that more variables may influence the patient’s recovery than chest tube management alone. Due to our retrospective design, the study relied on the accuracy of recorded data without upfront quality assurance, monitoring, control for missing data, nor retrieval of data for a formal cost-effectiveness analysis. While patients in the Netherlands are typically affiliated to a single hospital and therefore it is likely to return to the same hospital in case of a recurrence, patients may have been relocated during the study period, possibly affecting the reported recurrence rate. Despite lacking formal randomization, patients generally select their hospital based on residential proximity rather than the offered type of chest tube management. While this led to a merely random allocation based on geographical location, unidentified between-hospital differences may have acted as confounder, not all of which could be fully adjusted for.

## Conclusion

This multicentre retrospective comparative study demonstrates that progressive chest tube management after VATS for PSP significantly reduces LOS, chest tube duration, and complications without increasing the risk of recurrence ccompared to conservative management. These findings suggest that low-vacuum settings and prompt chest tube removal upon cessation of air leakage within 8 hours from surgery are both safe and effective. To validate our results and guide clinical practice, confirmation in a prospective randomized trial, which has already been initiated based on the findings of the present study (clinicaltrials.gov NCT06053476), is essential before early chest tube removal after VATS pleurodesis can be incorporated into national ERATS protocols.

## Supplementary Material

ezag136_Supplementary_Data

## Data Availability

The data underlying this article will be shared on reasonable request to the corresponding author.

## References

[1] Macduff A , Arnold A, Harvey J; BTS Pleural Disease Guideline Group. Management of spontaneous pneumothorax: British Thoracic Society pleural disease guideline 2010. Thorax. 2010;65:ii18-ii31. 10.1136/thx.2010.136986 20696690

[2] Batchelor TJP , Rasburn NJ, Abdelnour-Berchtold E, et al Guidelines for enhanced recovery after lung surgery: recommendations of the enhanced recovery after surgery (ERAS V) society and the European Society of Thoracic Surgeons (ESTS). Eur J Cardiothorac Surg. 2019;55:91-115. 10.1093/ejcts/ezy301 30304509

[3] Batchelor TJP. Enhanced recovery after surgery and chest tube management. J Thorac Dis. 2023;15:901-908. 10.21037/jtd-22-1373 36910059 PMC9992626

[4] Refai M , Brunelli A, Salati M, et al The impact of chest tube removal on pain and pulmonary function after pulmonary resection. Eur J Cardiothorac Surg. 2012;41:820-822; discussion 823. 10.1093/ejcts/ezr126 22219425

[5] Draeger TB , Gibson VR, Fernandes G, et al Enhanced recovery after thoracic surgery (ERATS). Heart Lung Circ. 2021;30:1251-1255. 33726996 10.1016/j.hlc.2021.01.014

[6] Feray S , Lubach J, Joshi GP, et al; PROSPECT Working Group of the European Society of Regional Anaesthesia and Pain Therapy. PROSPECT guidelines for video-assisted thoracoscopic surgery: a systematic review and procedure-specific postoperative pain management recommendations. Anaesthesia. 2022;77:311-325. 10.1111/anae.15609 34739134 PMC9297998

[7] Spaans LN , van Steenwijk QCA, Seiranjan A, et al Pain management after pneumothorax surgery: intercostal nerve block or thoracic epidural analgesia. Interdiscip Cardiovasc Thorac Surg. 2023;37:1–10. 10.1093/icvts/ivad180 PMC10645434 37941433

[8] Igai H , Matsuura N, Numajiri K, et al Feasibility of tubeless thoracoscopic bullectomy in primary spontaneous pneumothorax patients. Gen Thorac Cardiovasc Surg. 2023;71:138-144. 10.1007/s11748-022-01869-5 36036321

[9] Furuya T , Ii T, Yanada M, et al Early chest tube removal after surgery for primary spontaneous pneumothorax. Gen Thorac Cardiovasc Surg. 2019;67:794-799. 10.1007/s11748-019-01094-7 30798488

[10] Takamori S , Ebana H, Nakatsuka M, et al Evaluation of the safety of drainless uniportal video-assisted thoracoscopic surgery for the treatment of primary spontaneous pneumothorax: a two-institution retrospective study. J Thorac Dis. 2024;16:6537-6544. 10.21037/jtd-24-972 39552897 PMC11565299

[11] van Steenwijk QCA , Spaans LN, Heineman DJ, et al Variation in perioperative care for recurrent primary spontaneous pneumothorax: a dutch survey. Ann Laparosc Endosc Surg. 2023;8:9. 10.21037/ales-22-67

[12] Seely AJE , Ivanovic J, Threader J, et al Systematic classification of morbidity and mortality after thoracic surgery. Ann Thorac Surg. 2010;90:936-942; discussion 942. 10.1016/j.athoracsur.2010.05.014 20732521

[13] Digitale JC , Martin JN, Glymour MM. Tutorial on directed acyclic graphs. J Clin Epidemiol. 2022;142:264-267. 10.1016/j.jclinepi.2021.08.001 34371103 PMC8821727

[14] Twisk JWR. Inleiding in de Toegepaste Biostatistiek. 3rd ed. Amsterdam: Reed Business Education; 2014:13-340.

[15] Chan YH. Biostatistics 201: linear regression analysis. Singapore Med J. 2004;45:55-61. 14985842

[16] Sepehripour AH , Farid S, Shah R. Is routine chest radiography indicated following chest drain removal after cardiothoracic surgery? Interact CardioVasc Thorac Surg. 2012;14:834-838. 10.1093/icvts/ivs037 22392935 PMC3352714

[17] Holbek BL , Christensen M, Hansen HJ, et al The effects of low suction on digital drainage devices after lobectomy using video-assisted thoracoscopic surgery: a randomized controlled trial. Eur J Cardiothorac Surg. 2019;55:673-681. 10.1093/ejcts/ezy361 30445572

[18] Wagner G , Asban A, Xie R, et al Early water seal of chest tubes following video-assisted thoracic surgery pleurodesis. J Surg Res. 2023;283:1033-1037. 10.1016/j.jss.2022.11.052 36914993

[19] van Steenwijk QCA , Spaans LN, Heineman DJ, et al Population-based study on surgical care for primary spontaneous pneumothorax. Eur J Cardiothorac Surg. 2024;65:1-7. 10.1093/ejcts/ezae104 PMC10980590 38489837

[20] Sim SKR , Nah SA, Loh AHP, et al Mechanical versus chemical pleurodesis after bullectomy for primary spontaneous pneumothorax: a systemic review and meta-analysis. Eur J Pediatr Surg. 2020;30:490-496. 10.1055/s-0039-1697959 31600803

[21] Sudduth CL , Shinnick JK, Geng Z, et al Optimal surgical technique in spontaneous pneumothorax: a systematic review and meta-analysis. J Surg Res. 2017;210:32-46. 10.1016/j.jss.2016.10.024 28457339

