# Supplementary material for: Progressive Versus Conservative Chest Tube Management Following Surgery for Primary Spontaneous Pneumothorax
Source: Eur J Cardiothorac Surg. 2026 Mar 26;68(4):ezag136. doi: 10.1093/ejcts/ezag136 (PMC13106895; doi:10.1093/ejcts/ezag136)
Supplement: ezag136_Supplementary_Data [file ezag136_supplementary_data.docx]

**Supplemental material**

Progressive versus conservative chest tube management following surgery for primary spontaneous pneumothorax

**Supplementary Figure S1A-C. Directed acyclic graph illustrating known or potential causal relationships for all outcome measures**

**Chest tube management**

**LOS**

Hospital

Postoperative chest x-ray^a^

Thopaz setting^b^

Postoperative chest tube duration

Complications

Pleurodesis technique^b^

Age^b^

Postoperative pain

Mobility

**A**

PAL^b^

Surgical indication^b^

**Chest tube management**

**Complications**

Pleurodesis technique^b^

Bladder catheter^b^

Age^b^

BMI^b^

ASA^b^

Smoking^b^

Postoperative pain

Chest tube duration

Mobility

**B**

**Chest tube management**

**Recurrence**

Postoperative chest tube duration

Smoking^b^

Pleurodesis technique^b^

Age^b^

**C**

1A-C demonstrates the causal or non-causal paths between the exposure chest tube management and A) length of stay (LOS) and B) Complications yes/no and C) Recurrence yes/no. Variables with ^a^ are tested for confounding and ^b^ are tested for effect modifications. PAL: persistent air leakage, BMI: Body mass index, ASA: American Association of Anesthesiologists
The reported graphs are based on current clinical knowledge and may be updated in future studies, taking the results of this study into account.

**Supplementary table S1. Regression coefficients, 95%-confidence intervals and p values concerning the relation between chest tube management and outcome measures**

|  | β | | 95%-CI | P value |
| --- | --- | --- | --- | --- |
| Natural logarithm of LOS* | | | | |
| Chest tube management | -0.634 | | -0.805 to -0.462 | <0.001 |
| *Confounding* | | | | |
| Chest tube management | -0.370 (∆42%^1^) | | -0.542 to -0.198 | <0.001 |
| Chest x-ray | 0.638 | | 0.449 to 0.826 | <0.001 |
| *Effect modification* | | | | |
| Chest tube management | -0.616 | | -0.754 to -0.478 | <0.001 |
| PAL | 0.487 | | -0.295 to 1.269 | 0.221 |
| Interaction management*PAL | 0.662 | | 0.133 to 1.191 | 0.014 |
|  |  | |  |  |
| Chest tube management | -0.604 | | -1.110 to -0.098 | 0.020 |
| Thopaz setting | -0.001 | | -0.082 to 0.080 | 0.977 |
| Interaction management*chest tube setting | -0.015 | | -0.064 to 0.034 | 0.548 |
|  |  | |  |  |
| Chest tube management | -0.503 | | -0.876 to -0.129 | 0.009 |
| Age | 0.007 | | -0.001 to 0.014 | 0.074 |
| Interaction management*age | -0.004 | | -0.014 to 0.006 | 0.453 |
|  |  | |  |  |
| Chest tube management | -1.716 | | -2.351 to -1.081 | <0.001 |
| Pleurodesis technique | -1.342 | | -2.078 to -0.605 | <0.001 |
| Interaction management*pleurodesis technique | 0.951 | | 0.400 to 1.501 | 0.001 |
|  |  | |  |  |
| Chest tube management | -0.584 | | -0.796 to -0.371 | <0.001 |
| Surgical indication | 0.253 | | -0.322 to 0.827 | 0.387 |
| Interaction management*surgical indication | -0.055 | | -0.425 to 0.315 | 0.770 |
| Complications** | | | | |
|  | B | Exp(B) |  | |
| Chest tube management | -1.003 | 0.367 | 0.155 to 0.867 | 0.022 |
| *Effect modification* | | | | |
| Chest tube management | -0.811 | 0.444 | 0.095 to 2.081 | 0.303 |
| Smoking | 0.325 | 1.385 | 0.088 to 21.831 | 0.828 |
| Interaction management*smoking | -0.215 | 0.806 | 0.124 to 5.231 | 0.821 |
|  |  |  |  |  |
| Chest tube management | -1.132 | 0.322 | 0.047 to 2.232 | 0.252 |
| Age | 0.021 | 1.021 | 0.955 to 1.091 | 0.540 |
| Interaction management*age | 0.003 | 1.003 | 0.960 to 1.048 | 0.895 |
|  |  |  |  |  |
| Chest tube management | 15.731 | 6789156 | - | 0.999 |
| Pleurodesis technique | 14.969 | 3168273 | - | 0.999 |
| Interaction management*pleurodesis technique | -16.969 | - | - | 0.999 |
|  |  |  |  |  |
| Chest tube management | -0.893 | 0.409 | 0.019 to 8.900 | 0.570 |
| ASA | 0.821 | 2.274 | 0.275 to 18.833 | 0.446 |
| Interaction management*ASA | -0.051 | 0.950 | 0.213 to 4.238 | 0.946 |
|  |  |  |  |  |
| Chest tube management | -0.078 | 0.925 | 0.008 to 105.879 | 0.974 |
| BMI | 0.100 | 1.105 | 0.797 to 1.533 | 0.548 |
| INT_BMI | -0.045 | 0.956 | 0.768 to 1.191 | 0.691 |
| Recurrence** | | | | |
|  | B | Exp(B) |  | |
| Chest tube management | -0.536 | 0.585 | 0.179 to 1.917 | 0.376 |
| *Effect modification* | | | | |
| Chest tube management | -0.647 | 0.524 | 0.080 to 3.413 | 0.499 |
| Smoking | -0.701 | 0.496 | 0.013 to 19.197 | 0.707 |
| Interaction management*smoking | 0.148 | 1.159 | 0.102 to 13.126 | 0.905 |
|  |  |  |  |  |
| Chest tube management | -2.683 | 0.068 | 0.004 to 1.278 | 0.073 |
| Age | -0.132 | 0.876 | 0.738 to 1.041 | 0.132 |
| Interaction management*age | 0.072 | 1.075 | 0.978 to 1.182 | 0.135 |
|  |  |  |  |  |
| Chest tube management | -0.051 | 0.951 | 0.019 to 46.829 | 0.980 |
| Pleurodesis technique | 1.605 | 4.976 | 0.094 to 262.872 | 0.428 |
| Interaction management*pleurodesis technique | -0.056 | 0.946 | 0.054 to 16.410 | 0.969 |

LOS: length of stay, CI: confidence interval, PAL: persistent air leakage, β: regression coefficient, *linear regression analysis is used, **logistic regression analysis is used. ^1^The difference between unadjusted and adjusted β of progressive chest tube management.

**Supplementary table S2. Different scenarios with progressive or conservative chest tube management reflected on postoperative length of stay**

|  | **Chest tube management** | |  |
| --- | --- | --- | --- |
| **Different patient scenarios** | **Conservative**  N=84 | **Progressive**  N=99 | **Benefit**  (progressive - conservative) |
|  | **LOS (days)** | | |
| Average patient | 3.30 | 2.26 | -1.03 |
| *Effect on LOS per variable* | | | |
| talcage | 3.38 | 2.12 | -1.25 |
| pleurectomy | 2.88 | 3.25 | +0.37 |
| no PAL | 3.05 | 2.02 | -1.04 |
| PAL | 8.92 | 10.18 | +1.26 |
| postoperative chest x-ray | 3.74 | 2.57 | -1.17 |
| no postoperative chest x-ray | 2.44 | 1.67 | -0.76 |
| *Effect on LOS with multiple variables* | | | |
| Talcage, chest x-ray, no PAL | 3.55 | 2.15 | -1.40 |
| Talcage, chest x-ray, PAL | 10.36 | 10.85 | +0.49 |
| Talcage, no chest x-ray, no PAL | 2.31 | 1.40 | -0.91 |
| Talcage, no chest x-ray, PAL | 6.75 | 7.06 | +0.32 |
| Pleurectomy, chest x-ray, no PAL | 3.03 | 3.29 | +0.26 |
| Pleurectomy, chest x-ray, PAL | 8.83 | 16.59 | +7.76 |
| Pleurectomy, no chest x-ray, no PAL | 1.97 | 2.14 | +0.17 |
| Pleurectomy, no chest x-ray, PAL | 5.75 | 10.80 | +5.06 |

This table presents the length of stay (LOS) under conservative and progressive chest tube management across different patient scenarios. The scenarios are defined by the variables identified as a confounder (chest X-ray findings: yes [1]/no [0]; mean score: 0.705) and as effect modifiers (pleurodesis technique [talcage [1] vs pleurectomy [2]; mean score: 1.148] and presence of persistent air leak [PAL]: yes [1]/no [0]; mean score: 0.071). The table serves to illustrate the impact of these variables on LOS. It should be noted that, as multiple variables are combined, the number of patients within each scenario decreases, which may result in less reliable estimates.
